# Testing the psychometric properties of a short skills inventory for students looking for their first job

**DOI:** 10.1186/s40359-021-00662-y

**Published:** 2021-10-18

**Authors:** Rosa Isabel Rodrigues

**Affiliations:** 1 Instituto Superior de Gestão, Avenida Marechal Craveiro Lopes, no 2a, 1700-284 Lisbon, Portugal; 2 The Transdisciplinary Research Center of Innovation and Entrepreneurship Ecosystems (TRIE), Universidade Lusófona de Humanidades E Tecnologias, Campo Grande, no 376, 1749-024 Lisbon, Portugal

**Keywords:** Human resources, Psychological assessment, Construction and validation, People selection, Skills

## Abstract

**Background:**

In the last two decades, the transformations that have affected the business world have had a great impact on professional performance standards. As such, they have contributed significantly to increasing concerns regarding employability. Particularly, these concerns are even more worrying among students who are looking for their first job. Consequently, this leads organizations to question whether the skills these candidates have are sufficient and adequate for them to enter the job market. Although it is a problem that deserves an urgent response, it is still poorly understood amongst academics, which is why it continues to be essential to define and signal which skills candidates should develop in order to guarantee a better person-function fit. Hereupon, the present study aims at the construction and validation of a short skills inventory for students who are looking for their first job. This inventory will allow alignment between candidates' skills and the level of performance expected by their future employers.

**Methods:**

The development of the short skills inventory for students looking for their first job was based on Classical test theory and Item response theory methodologies. Specifically, its developmental process encompassed three studies. Study 1, comprising a qualitative scope, dealt with the development and construction of the items (n = 97). Study 2, of an exploratory nature, was intended to evaluate the psychometric properties of the instrument (n = 173). Finally, Study 3, of a confirmatory nature, was aimed at validating the results gathered from the Exploratory factor analysis (n = 407).

**Results:**

This inventory is a valuable asset for the selection of students who are looking for their first job. The analyzes carried out over the various studies show that this instrument has satisfactory psychometric properties, and, as such, is a valid and reliable instrument and an alternative to the instruments currently used in the recruitment and selection processes.

**Conclusions:**

The construction of this short skills inventory brings theoretical and practical benefits. In short, it contributes to reducing Portugal’s gap regarding psychological assessment upon selection considering the lack of validated and verified instruments for students looking for their first job.

## Background

The new macroeconomic realities presented by a globalized world are changing the labor market and making it increasingly competitive. To respond positively to this reality, organizations need professionals who are able to get involved with the organization's goals and also implement changes that allow them to achieve a competitive advantage [1]. However, access to labor is challenging for recent graduates who, despite being the main candidates in the selection processes, are a group that is often underestimated in terms of research [2].

In such dynamic environments, in order to promote the development of the skills that these individuals need, organizations have to stand out through sustainability [3], not just due to their quality or level of productivity [4] but the skills they are able to structure regarding their means of acting. In the long run these are considered as performance outputs seen as distinctive marks held by successful organizations [5]. To proceed to selection of the best candidates, evidence is also necessary regarding the skills that are most valued by their future employers [6]. In short, in doing this, both candidates and employers are informed about what to expect.

In order to fill the gap that has been pointed out by several authors (e.g., [2, 7, 8]) the present study is aimed at developing and validating a short skills inventory for students who are looking for their first job. In practice this will allow an answer to the question regarding which skills are essential to align a candidate's profile with an employer’s expectations [9]. But how can we fulfil this need?

Bartram’s [10] Great Eight Model allows this need to be theoretically met. Specifically, the factors that make up the model provide a unique framework for making predictions based on the assessment of the candidates' skills potential. It also has the advantage of allowing an individual to quickly and efficiently select the right person for the right place, as each of them predicts a different area of performance [11]. In addition, since its inception, it has been used to create 403 skills models that have allowed large companies to stand out amongst their organizational competitors [12].

Additionally, and in practice, a sample of senior managers helps in identifying the skills that are most valued in professional practice [6]. As such, the inventory considers the skills privileged by the employer, as it is important to know which skills, they value most in a specific work context, and which will allow them to improve their employability indexes. Also, its computerized format improves the recruitment and selection processes, making them more efficient, faster, and less expensive. Furthermore, it avoids the inconvenience associated with errors inherent to the administration and quotation of tests [13].

## Skills

In the last two decades, the transformations that have been affecting the business world have had a great impact on professional performance standards, which as a result contributed significantly to increasing concerns regarding employability [14]. Particularly, these concerns are even more worrying among students who are looking for their first job and as a consequence it leads organizations to question whether their skills are sufficient and adequate to enter in the labor market [15]. Although it is an issue that deserves an urgent response, it is still poorly understood amongst academics [7], which is why it continues to be essential to define and signal which skills candidates should have in order to guarantee a better person-function fit [6].

However, before continuing, it is important at this point to clarify the origin of the concept of competence so that we can understand its importance in the development and application of the concept of soft skills amongst students who are looking for their first job.

The concept of competence^1^ came to light in the early 1970s, with the publication of *Testing competence rather than intelligence* by David McClelland [16], which questions the efficiency of personality tests and the academic path of candidates by considering that such factors represented a disadvantage for minorities, women and people with a lower socioeconomic status. Such conclusions led him to investigate the differences between outstanding performers and those whose performance was just enough to allow them to keep their jobs. His research allowed him to discover that people are not differentiated by their skills profile, but instead by the actual results of their performance. The author suggests the pursual of a process that allows the level of performance to be predicted more reliably by using skills assessment [17].

Over the years, McClelland’s work has been complemented by several authors (e.g., [10, 18]) and, although the concept of skill has been one of the most used in the organizational context over the last decades and constantly present in this field of literature, the concept remains without a simple definition to this day [19]. However, its status as an essential tool to help professionals achieve the desired results [20] is commonly agreed upon. Based on these considerations, Table 1 presents some definitions that have emerged over time, according to the perspective of authors who have dedicated themselves to the study of this subject.

**Table 1:** Evolution of the concept of skill

Author(s)/Year	Definition of the concept of skill
McClelland [16]	Element capable of predicting the performance of individuals, regardless of race, gender or socioeconomic factors
Spencer and Spencer [43]	Deep and structured part of the personality that can predict behavior in a wide variety of situations or professional activities
Prahalad and Hamel [101]	Ability to combine, mix and integrate resources in products and services
Fleury and Fleury [102]	Knowledge and ability to act that must be aligned with organizational strategy and resources
Bücker and Poutsma [103]	Abilities, knowledge, attitudes and behaviors that allow you to perform specific tasks effectively
Bartram [10]	Set of fundamental behaviors to achieve organizational goals
Taylor and Bond [18]	Combination of knowledge, aptitudes, abilities and personal characteristics necessary for effective performance
Rutledge et al. [104]	Specific behaviors evidenced with a certain constancy and regularity in the exercise of different professional activities
Ng and Kee [105, p. 255]	Set of knowledge, attitudes and behaviors that make it possible to recognize and take advantage of opportunities and create ways to achieve them
LeCompte et al. [106]	Aggregation of knowledge, aptitudes and values that encompass communication, problem solving, critical and creative reflection and decision making

Through analysis of Table 1 and according to Ceitil [19], the multiple definitions applied to the concept of skill can be conceptualized from four different perspectives: (1) attributions, (2) qualifications, (3) personal traits or characteristics, and (4) behaviors or actions. Skills, in the attributions scope, are considered as an exogenous factor, pertaining rather to certain advantages that are connected to specific positions, functions or responsibilities and not to someone’s characteristics and performance; in the qualifications scope, skills refer to a set of knowledge/mastery of technical execution that can be acquired through formal education or professional training systems; when considering skills as traits or personal characteristics, we define what the person is according to their actions, i.e., that superior performance in a given activity derives directly from someone’s intrinsic characteristics; and, finally, when skills are placed as being related to behaviors or actions, they pertain to a person’s ability and potential to successfully perform certain tasks [21].

It also becomes clear that skills encompass three dimensions: (1) the cognitive, that includes systemic thinking and pattern recognition; (2) the emotional, that relates to self-confidence, self-control, adaptability, positive vision and orientation for results; and (3) the social, that involves empathy, organizational awareness, influence, conflict management, teamwork, support and development, and inspirational leadership. These premises are in line with the studies developed by Amdurer et al. [22]. According to these authors, skills that are connected to cognitive intelligence are strongly correlated with systemic thinking and pattern recognition,those skills related to social intelligence can be associated to teamwork, negotiation and empathy; and the skills associated with emotional intelligence (e.g., emotional awareness and self-control) correlate with adaptability and results-orientation. The authors state that self-confidence, initiative and orientation for achievement can act as a predictive factor regarding job satisfaction and success.

Krumm et al. [23] add that skills models are crucial for decision-making (e.g., staff planning, recruitment, training, promotions, remuneration), so it is not surprising that they are considered extremely important in organizations’ day-to-day reality.

### Soft skills

Considering this, organizations are starting to look for professionals who present transversal and differentiating attitudes, behaviors and competencies—commonly referred to as soft skills. Notwithstanding their importance, technical skills are no longer sufficient, per se, to ensure a prominent place in the labor market [24]. Some soft skills that organizations seem to value the most are leadership, teamwork, critical thinking, logical reasoning, communication, holistic thinking, assertiveness, creativity, orientation for results and negotiation [20]. These will be briefly defined.

Those who decide, take initiatives, assume responsibilities, who execute and take calculated risks, guide and coordinate activities, supervise, delegate, attribute responsibility and motivate others [25] can be characterized as showing leadership skills. Teamwork, in turn, is fundamental to increased productivity, as the structures that allow for learning, for change and—consequently—for competitive advantage are created through sharing knowledge, abilities, attitudes, behaviors and motivation among the group members [26]. Critical thinking is a quality of individuals that spot inconsistencies and solve problems in a systematic way [27]. Logical reasoning defines people who have the capacity to determine a conclusion by applying rules that include deduction, induction and abduction [28]. Communication skills are related to the ability to express ideas in a clear and objective manner [29].

On another note, Deepa and Seth [30] point that only those who are always eager to learn more and who show good communication abilities and ease in acquiring new contacts can prosper and achieve success. Those who understand that the whole is more than the mere sum of the parts can be characterized by their holistic thinking [31]. Assertiveness is a quality shown by professionals who have the ability to control what goes on in social gatherings and that have great tolerance for criticism—whether positive or negative [32].

Creativity describes the skills that are used in the creation, invention, discovery and development of good ideas and also in solving new problems, aiming to achieve the organization's goals [33]. Regarding orientation towards results, Dubey and Ali [34] state that it characterizes a professional who focuses on achieving objectives to ensure that they are accomplished, and who is fully aware of its priorities whilst being persistent regarding overcoming obstacles and adversities that may arise. Finally, negotiation concerns the ability to remediate conflicts and reconcile interests [35].

Although resilience is not amongst the ten most-valued skills in Gabor et al. [36], 42.0% of the employers mentioned it, suggesting that the ability to overcome obstacles and frustrations seems to be highly relevant in the organizational context, particularly regarding career development and management. Furthermore, the job market values people who can easily adapt to new contexts and know how to uphold a positive outlook, because—aside from influencing colleagues with their energy, joy and enthusiasm—those persons seem to be more productive and determined when pursuing a goal [37]. Also, organizations want professionals who are able to understand and solve problems efficiently, effectively and promptly, which shows that time management is another key competence needed for achieving professional success [38]. Brown et al. [39] further adds that the ability to define goals, identify priorities, and plan and organize work are some of the requirements that have become increasingly valued by organizations, as they are essential for responding to the demands presented by the current market.

Skills have become an integral part of people management. To allow for their effective channeling, it is necessary to assess skills according to each person's abilities, development needs and potential [40]. In this context, four large sets of variables can be defined: (1) the behaviors used to achieve specific objectives; (2) the potential and competencies that are influenced by personal attributes (e.g., abilities, interests, values, motives, personal style, knowledge and/or skills; (3) the requirements that link the potential to that particular competence; and (4) the results defined by the individual, hierarchical superiors or by the organization itself [41].

### Skills models

**Fig. 1:**
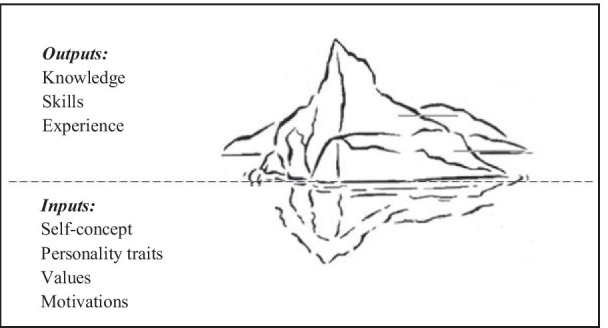
Iceberg model. ( Adapted from [43])

In light of the foregoing, it appears that skills models are essential to identify which abilities are necessary to perform a specific task or role in an organization, with successful companies being defined as those who know how to take advantage of their employees’ potential and manage their individual skills [42]. Spencer and Spencer’s [43] model considers the existence of two types of skills, that can be represented as an iceberg (Fig. 1). The dimension above surface corresponds to the knowledge applied and to the expertise shown through technical knowledge, whereas the submerged dimension concerns the employees’ personal characteristics, attitudes, values and motivation. This model proposes the existence of a divisional line between internal and external skills [19].

The skills that are submerged encompass motivations, personality traits, self-concept and self-values, deeply rooted in the person. On an individual’s surface, the skills that can be found are knowledge and experience that correspond to the outputs shown by someone when he performs. In other words, what ultimately makes the difference is what is on the surface of the iceberg. It is at this level that high-performance employees can be observed and identified, and where one can act in order to develop the necessary skills to achieve the organization's goals [44]. In this model, skills emerge as the subject’s underlying characteristics, having a cause-effect relationship with the average or superior performance of a function, which contrasts with the later perspectives, especially with the notion of skill and its relationship with the work context, as seen in the following models [21].

The Great Eight Model, developed by Bartram [45], allows organizational performance and effectiveness to be predicted through the assessment of observable behavior. According to the author, skills correspond to a set of observable actions, which may include personality characteristics, aptitudes, motivations and interests. This model is based on the Universal Competency Framework [46], the foundations of which lie on the following assumptions: (1) the work context presents quantitative and not qualitative differences, so skills can be defined using the same dimensioning, regardless of the dominant country or culture; (2) it is possible to identify key behaviors and skills components for different types of functions and goals and; (3) the contents of any model should not be completely fixed, as its elements may change over time.

**Fig. 2:**
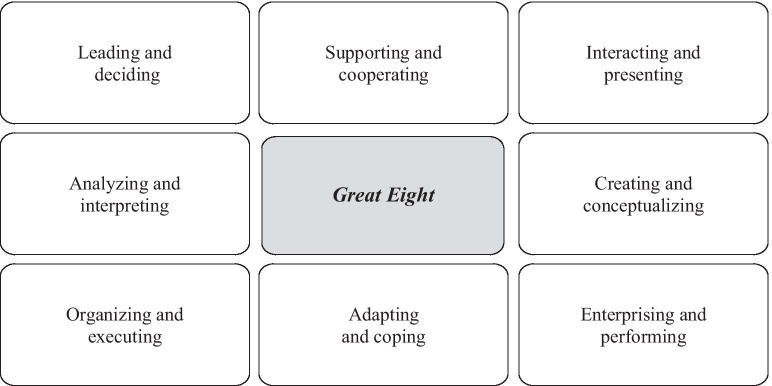
Great eight model. ( Adapted from [10], p. 7)

Considering that this is a triarchic and, therefore, multifactorial model, its development starts with a set of studies regarding the factor and multidimensional analysis of performance evaluation scales, whose results gave rise to 112 competence components distributed over 20 skills, which are then aggregated into eight higher-level factors (Fig. 2): (1) leading and deciding, (2) supporting and cooperating, (3) interacting and presenting, (4) analyzing and interpreting, (5) creating and conceptualizing, (6) organizing and executing (7) adapting and coping and (8) enterprising and performing [47].

The first factor—leading and deciding—includes the following dimensions: ‘deciding and taking initiative’ and ‘leadership and supervision’, which are being related to the ability to organize and manage individuals and/or teams by making use of the employees’ potential, by motivating and involving them and by defining tangible objectives that converge towards the organization's goals, with the aim of obtaining results and enhancing employees’ skills [48].

The supporting and cooperating factor characterizes a person who understands and supports others, builds team spirit, recognizes and rewards employees, knows how to listen, communicates proactively, shows empathy, tolerance and consideration, follows principles and values, and whose actions show integrity, and social and environmental responsibility. This skill encompasses the components: ‘working with people’ and ‘following principles and values’. According to Raina and Zameer [49] communication skills are essential in this domain, as it is through them that one can identify the needs of the employee and understand his points of view.

Factor three—interacting and presenting—includes relationship and networking skills, persuading and influencing, and presenting and transmitting information. It is used to describe a person who knows how to manage conflicts, negotiate, argue, pass information along easily and also shows great credibility. Wei et al. [50] report that, despite the effort invested in creating and maintaining professional networks—especially when there is a transfer of complex knowledge—these have a significant correlation with job performance.

The components ‘writing and reporting’, ‘applying expertise and technology’, and ‘analyzing’ are part of the fourth factor—analyzing and interpreting. People who write clearly and fluently, know how to develop and apply technical knowledge, make use of technological resources, share knowledge, analyze and evaluate information, investigate and test hypotheses, present solutions and have systemic thinking can be included in this domain. According to Foster et al. [51], the business environment status quo requires organizations to analyze and interpret large volumes of information in order to ensure better and more informed decision-making. Anderson et al. [52] further adds that the skills that concern written and spoken communication are essential for professional success.

The creating and conceptualizing factor comprises the dimensions ‘learning and researching’, ‘creating and innovating’, and ‘formulating strategies and concepts. This dimension characterizes a person who learns and thinks quickly, knows how to collect information and manage knowledge, establishes and develops work strategies and thinks holistically [29].

The sixth factor—organizing and executing—describes a person who knows how to plan, organizes and sets goals, manages time and resources, monitors progress, meets customer expectations, sets high quality standards, works systematically, maintains levels of productivity, and is committed to the organization. This factor includes the components ‘planning and organizing’, ‘obtaining results and meeting customer expectations’, and ‘following instructions and procedures’ [46].

Factor seven—adapting and coping—involves one’s ability to adapt to changes and to deal with pressure and setbacks. People with higher marks in this dimension adapt easily to change, accept new ideas, demonstrate intercultural awareness and emotional self-control, know how to deal with ambiguity and are able to balance work and personal life. Eisenberg et al. [53] state that this skill is not only desirable but essential, as the number of people with different values and cultures who interact daily in the work context has grown substantially due to the ever-increasing globalization processes that have been emerging in recent years. This situation has made it imperative for professionals to develop skills that make them culturally skilled to deal with diversity, in order to understand how the different national cultures can influence the organizations’ dynamics.

Finally, enterprising and performing describes people who achieve their personal and professional goals and objectives, work energetically and enthusiastically, are ambitious, have a business and commercial-oriented mind, follow markets and competition attentively and know how to identify business opportunities. This factor aggregates the components: ‘meeting personal and professional goals and miles’, and ‘business and commercial thinking’. When behaviors oriented to this domain are displayed, this can be related to a continuous search for new knowledge that is both relevant to the professional activity and addresses the market demands [46].

It should be noted that these factors would be defined and evaluated according to the work context in which they are employed, so the choice regarding how to assess them depends on a set of material and human resources and on the organization's goals [19]. When using this type of instruments, there are no right or wrong answers—the sole intention is to ascertain opinions, behaviors and attitudes towards any given situation [54].

## Overview of the research design

The aim of the present study is to develop and validate a short skills inventory for students who are looking for their first job. To do so, Bartram’s [10] line of work will be followed,specifically, his Great Eight Model. As such, it is postulated that the eight factors are correlated and come together to define the candidate's profile regarding their skills, in order to align them with the employer's expectations. In order to corroborate this, the research design is based on the study of the Great Eight Model’s perceived utility, factorial structure and internal consistency. Additionally, the model’s convergent and discriminant validity will be tested.

In detail, the development and validation of the short skills inventory for students who are looking for their first job will take place over the course of three studies: the first, with a qualitative scope, aims at the development of the items based on the senior managers’ view of the skills that are perceived as most useful in practice; the second, of an exploratory nature, focuses on the analysis of the instrument's psychometric properties; and the third, which is confirmatory, aims to validate the results from the exploratory factor analysis (EFA) and confirm if the measured variables adequately represent the number of constructs obtained. It also intends to ascertain if the latent factors are responsible for the behavior of the manifest variables [55].

## Study 1. Development and construction of the items

For the development and construction of the items, 97 semi-structured interviews were conducted with the top management of a consulting company. Specifically, for the elaboration of the questions we resorted to the participation of the partners and managers that had most years of experience in the company.

The interviews included four questions: (1) what skills are needed to be part of the organization's executive staff; (2) what are the characteristics that distinguish top and bottom performers; (3) what skills could be developed/improved to solve daily problems; and (4) which skills need to be developed to obtain a high mark when they have to be evaluating regarding their performance. After collecting the data, the interviews were fully transcribed, to allow for their coding. To do so, the authors employed MAXQDA 11 software, following the content analysis steps suggested by Bardin [56], namely: (1) pre-analysis, (2) material exploration and (3) treatment of results, inference and interpretation.

According to Succi and Canovi [6], employers must play an active role in the recruitment and selection process and its preparation, particularly regarding the requirements that candidates must have, as they are the ones who best know the skills necessary in order for the candidates to better perform in their future function [7]. Testimonials like the ones that follow show this need:

“*When recruiting new candidates, we expect them to act with integrity and show empathy for others in order to stimulate team spirit*.”^2^

“*Essentially we expect them to think outside the box, because only then can they identify new business opportunities and achieve the proposed goals*.”^3^

Analyzing the content of the interviews was important in identifying the most-valued skills in a work context, and also in selecting a theoretical model that would allow for their evaluation, where the Great Eight proved to be the one that best suited the answers provided (Table 2).

**Table 2:** Components, skills and factors most valued by employers

Skills components	%	Skills	%	Factors	%
Decision-making	1.0	Deciding and taking initiative	2.0	Leading and deciding	8.1
Act with confidence	1.0				
Delegate	5.6	Leadership and supervision	6.1		
Motivating others	0.5				
Build team spirit	3.9	Working with people	10.9	Supporting and cooperating	14.6
Communicating proactively	4.8				
Show empathy	0.6				
Supporting others	1.6				
Preserving ethics and values	2.3	Following principles and values	3.7		
Act with integrity	1.5				
*Networking*	1.5	Relationship and networking	2.8	Interacting and presenting	7.3
Conflict management	1.3				
Negotiation	1.5	Persuading and influencing	1.5		
Explain concepts and opinions	3.0	Presenting and transmitting information	3.0		
Write correctly	1.8	Writing and reporting	3.0	Analyzing and interpreting	17.2
Communicate in order to reach a specific target	1.2				
Apply technical knowledge	4.5	Applying expertise and technology	12.4		
Analyze and evaluate information	1.9	Analyzing	1.9		
Test hypotheses and investigate	2.7				
Present /produce solutions	1.9				
Ability to give opinion	1.5				
Learn quickly	3.0	Learning and researching	4.5	Creating and conceptualizing	10.1
Speed of thought	1.5				
Innovate	3.7	Creating and innovating	3.7		
Think holistically (contemplate the whole)	0.8	Formulating strategies and concepts	1.9		
Be visionary	1.1				
Plan	4.8	Planning and organizing	10.7	Organizing and executing	26.1
Time Management	5.8				
Focus on customer needs and satisfaction	4.2	Obtaining results and meeting customer expectations	9.6		
Monitor and maintain quality	1.9				
Work systematically	4.1				
Maintain the productivity levels	3.5				
Follow procedures	1.8	Following instructions and procedures	5.9		
Commitment	4.1				
Adaptation	3.9	Adapting to change	6.2	Adapting and coping	9.4
Dealing with ambiguity	2.3				
Dealing with ambiguity	2.6	Dealing with pressure and setbacks	3.2		
Work-life balance	0.5				
Reach goals	1.9	Meeting personal and professional goals and miles	5.8	Enterprising and performing	7.2
Self development	5.8				
Identify business opportunities	1.3	Business and commercial thinking	1.3		

As such, 120 items were developed, 15 for each of the eight dimensions: (1) leading and deciding, (2) supporting and cooperating, (3) interacting and presenting, (4) analyzing and interpreting, (5) creating and conceptualizing, (6) organizing and executing, (7) adapting and coping, and (8) enterprising and performing.

**Fig. 3:**
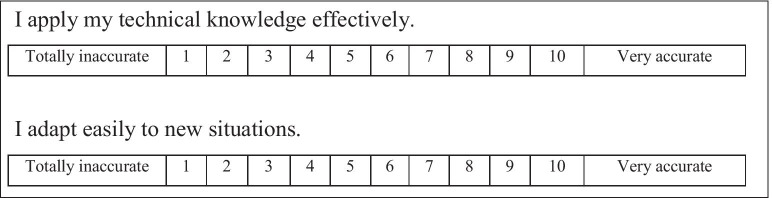
Evaluation of skills items

The items were evaluated on a 10-point Likert scale (1 = Totally inaccurate to 10 = Very accurate) and the score was calculated by adding the value given to the items that make up each of the dimensions (Fig. 3). According to Masters [57] and Weng [58] the internal consistency of the instrument increases as the number of response categories increases.

Subsequently, with the participation of ten academics from the psychological assessment and psychometry fields, a spoken reflection of the items was carried out. It should be noted that all of them have over eight years of professional experience (M = 19.8; SD = 8.87). This step allowed us to compile the experts' opinions and check their level of agreement regarding the items to include in the inventory. As such, items with a score equal to or greater than 75 (results greater than ¾ of responses, as recommended by Howell [59] were selected, in a total of 48 items (six per dimension). Before applying the inventory, the items were randomized, because randomness can help to reduce order bias and improve data quality [60].

## Study 2. Exploratory study

With the first application of the inventory, an attempt was made to test the adequacy of the items to the target audience and to analyze the participants' reaction to the questions.

### Method

#### Sample

The skills inventory was applied to 173 subjects, aged between 21 to 62 years (M = 32.05; SD = 11.41). It should be noted that 59.0% of participants were female, and all participants had attended higher education, with management (27.2%) and psychology (16.2%) being the most common among their fields of training.

#### Procedures

Data were collected in a classroom, after professors and students granted their consent. The participants were informed about the goals of the study, and also that their participation would be voluntary, thus allowing them to withdraw from the study at any point should they wish to do so. The confidentiality of the results was also guaranteed, further ensuring that the data would only be used in an academic context.

### Results

Due to the small sample size (less than five people per item; [61]) it was not possible to analyze all items simultaneously, which led to the decision to analyze only one dimension at a time. The relational structure of the items belonging to each skill was evaluated through the correlation matrix, with factors being extracted through principal components analysis (PCA) with varimax rotation, and from which the three highest-loading items in each dimension were extracted [62]. The aim of the option for the use of three items was to bring the number of items closer to what was desirable for the sample size and to reduce the length of the questionnaire [63].

A new factorial analysis was performed after this selection, thus allowing the authors to verify the adequacy of the model (KMO = 0.95) and the existence of an identity matrix in the data [*χ*^2^_(276)_ = 3806.170, *p* < 0.001].

The variance percentage explained for the eight skills extracted was at 83.24% (Table 3). After reordering items, the first factor regarding adapting and coping showed high factor weights for items 1, 2 and 3 and explains 17.46% of the total variance. The second component explains 12.49% of the results’ variance, being composed by items 4, 5 and 6, pertaining to analyzing and interpreting. The third component presents an explained variance of 11.52% and corresponds to supporting and cooperating, consisting of items 7, 8 and 9. The fourth component consists of items 10, 11 and 12, explains 10.75% of the total variance and concerns the creating and conceptualizing dimension. In fifth place, with 9.58% of variance, comes enterprising and performing, which encompasses items 13, 14 and 15. The sixth component joins items 16, 17 and 18, belonging to leading and deciding, and explains 7.42% of the variance. Component seven refers to organizing and executing and is responsible for 7.19% of the results’ variance, comprising items 19, 20 and 21. Finally, the eighth component concerns interacting and presenting relationships and shows an explained variance of 6.80% (items 22, 23 and 24) Additional inventory files shows this in more detail.

**Table 3:** Factor matrix of the skills inventory after varimax rotation

	Factor 1	Factor 2	Factor 3	Factor 4
1. I find it easy to adapt to new cultures	0.783			
2. I am able to adjust my behavior to different contexts	0.759			
3. I adapt easily to new situations	.626			
4. I quickly understand the new technologies related to my profession		0.789		
5. I usually compare information to check similarities, differences and congruities		0.779		
6. I continuously keep my professional knowledge up to date		0.767		
7. I usually support the people I deal with daily			0.824	
8. My co-workers can always count on me			0.771	
9. I often pass along my knowledge to foster team spirit			0.732	
10. I can quickly share the knowledge/information I have stored whenever I am asked				0.798
11. I see new situations as challenges to overcome				0.764
12. I try to develop ideas that can drive organizational change				0.548
*Eigenvalue*	14.96	3.12	2.87	1.66
% explained variance	17.46	12.49	11.52	10.75
*Cronbach alpha*	0.86	0.85	0.90	0.82
AVE*	0.52	0.58	0.60	0.50
CR*	0.76	0.80	0.81	0.75

	Factor 5	Factor 6	Factor 7	Factor 8
13. I know all areas of the organization I work for	0.773			
14. I often look for business opportunities in poorly dominated areas	0.704			
15. I’m always alert regarding any opportunities for personal development	0.673			
16. I foster the organization’s staff professional development ara>		0.866		
17. I motivate my team towards success		0.815		
18. I often take the initiative		0.779		
19. I can identify priority jobs			0.756	
20. I perform my tasks in an organized manner			0.696	
21. When conducting a meeting, I prepare the list of matters to be dealt with in advance			0.629	
22. I try to benefit both parties when establishing an agreement				0.767
23. I positively influence my co-workers				0.760
24. I find it very easy to relate to people of all hierarchical levels				0.651
*Eigenvalue*	1.35	1.13	1.06	1.01
% explained variance	9.58	7.42	7.19	6.80
*Cronbach alpha*	0.77	0.86	0.82	0.85
AVE*	0.51	0.68	0.48	0.52
CR*	0.72	0.86	0.73	0.77

Factor 1 = Adapting and coping; Factor 2 = Analyzing and interpreting; Factor 3 = Supporting and cooperating; Factor 4 = Creating and conceptualizing; *AVE and CR was calculated manually based on formula given by Fornell and David [64] and Valentini and Damásio [66]

Factor 5 = Enterprising and performing; Factor 6 = Leading and deciding; Factor 7 = Organizing and executing; Factor 8 = Interacting and presenting; *AVE and CR was calculated manually based on formula given by Fornell and David [64] and Valentini and Damásio [66]

Reliability was analyzed using Cronbach's alpha coefficient, which allows us to verify if each extracted component measures a single latent construct. As such, the higher the value of this index, the lower the influence of measurement errors and the greater the internal consistency of the items [55].

The results reveal that all subscales have adequate internal consistency: 0.86 for adapting and coping, 0.85 for analyzing and interpreting, 0.90 for supporting and cooperating, 0.82 for creating and conceptualizing, 0.77 for enterprising and performing, 0.86 for leading and deciding, 0.82 for organizing and executing and 0.85 for interacting and presenting. Composite reliability (CR) was used as measure of internal consistency of the factors, where values greater 0.70 indicate good reliability. To compute convergent and discriminant validity, Fornell and Larcker’s [64] procedures were used. According to these procedures, and Rebelo-Pinto et al. [65], discriminant validity is obtained if the average variance extracted (AVE) is greater than the maximum shared squared variance (MSV) or the average shared squared variance (ASV). Regarding the convergent validity, the AVE should be equal to or greater than 0.50 and lower than the CR. That is, the variance explained by the construct should be greater than the measurement error and greater than the cross-loadings.

The CR values were equal to or greater than 0.73 and the AVE was greater than 0.48, which is indicative of convergent validity. The results obtained through the Fornell and Larcker [64] procedures reveal that the average variance extracted (AVE), whose values range between 0.48 and 0.68, is greater than the maximum shared square variance (MSV = 0.67) and the average shared square variance (ASV = 0.43), which suggests that the eight factors have good internal consistency and convergent and discriminant validity [66]. The exploratory study proved to be useful as it allowed the adequacy and understanding to be checked, with regard to the instructions and scale used.

**Fig. 4:**
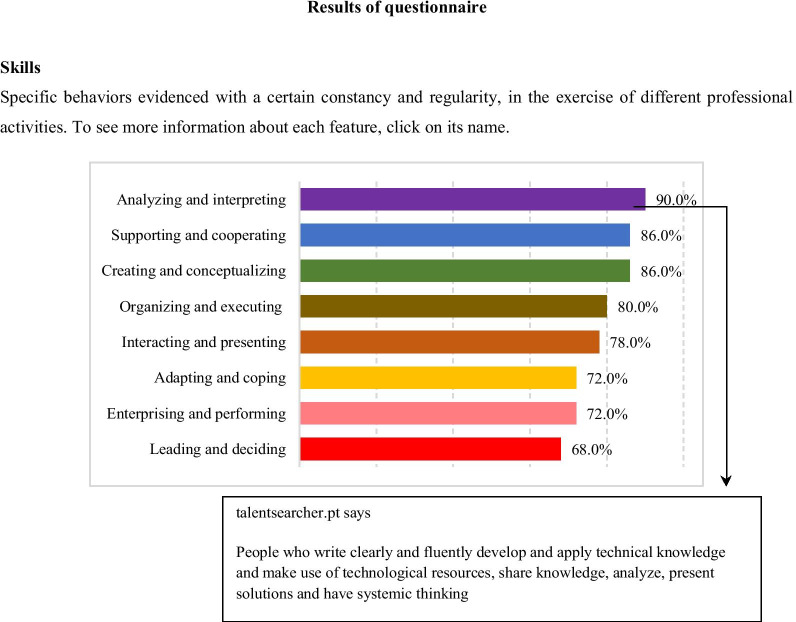
Example of a report

Participants were informed that a report (Fig. 4) would be available, through an e-mail address they had the option of providing if they wished to receive it. All the dimensions encompassed are accompanied by a brief description of what they evaluate. To see this, one simply needs to click on the dimension name, which will prompt a window with a more detailed explanation of each one of them.

The analysis of Questionnaire No. 202 shows that the skill that stands out the most is that of analysis and interpretation, which suggests that the subject can easily develop and apply technical knowledge, effectively use technological resources, analyze and present solutions, share knowledge, and write and report results clearly and fluently [46].

Finally, the association between the various skills was studied. It was found that they are all significantly correlated (Table 4). The correlations between the variables, with values ranging from 0.356 to 0.787, suggest that they are adequate to assess these skills. It was also found that the highest correlation is that between “supporting and cooperation” and “adapting and coping” (*r* = 0.787, *p* < 0.001). Both these skills are characterized by the ability to deal with people [67].

**Table 4:** Descriptive statistics and correlations for the great eight model skills

	*M*	*DP*	1	2	3	4	5	6	7
AI (1)	3.79	0.94	–						
SC (2)	4.30	0.85	0.503**	–					
IP (3)	5.05	0.98	0.500**	0.592**	–				
LD (4)	4.69	1.01	0.356**	0.486**	0.645**	–			
CC (5)	4.23	0.88	0.431**	0.550**	0.659**	0.709**	–		
OE (6)	4.08	0.78	0.395**	0.369**	0.406**	0.404**	0.470**	–	
AC (7)	4.70	0.99	0.534**	0.787**	0.595**	0.599**	0.676**	0.370**	–
EP (8)	3.57	0.88	0.501**	0.507**	0.588**	0.553**	0.485**	0.377**	0.544**

*M* Mean, *SD* Standard deviation; **p < 0.001, *SC* Supporting and cooperating, *AI* Analyzing and interpreting, *IP* Interacting and presenting, *LD* Leading and deciding; *CC* Creating and conceptualizing, *OE* Organizing and executing, *AC* Adapting and coping, *EP* Enterprising and performing

## Study 3. Confirmatory study

First, the results of the classical test theory (CTT) are presented, namely, the inventory of psychometric indicators. Secondly, the results of the item response theory (IRT), are presented though the use of Rasch models, focused on analyzing the dimensionality, Rasch item fit and item difficulty of the dimensions that compose the skills inventory.

### Method

#### Sample

This study had the participation of 407 students, attending both public and private higher education institutions, aged between 18 and 56 years (M = 24.8; SD = 7.09), 52.8% were women.

Due to the difficulties inherent to the logistics behind the test application (e.g., use of a computer) and to the constraints posed by the availability of professors from the institutions involved, a convenience sample was used. This study sought to cover the largest possible number of study areas: public administration (8.1%), architecture (5.4%), political science (1.5%), educational sciences (1.5%), communication (3.7%), accounting (2.5%), design (2.5%), law (1.7%), economics (6.4%), nursing (4.2%), biomedical engineering (1.2%), electronic engineering (3.4%), computer engineering (9.1%), European studies (2.9%), management (12.5%), information management (3.4%), sports management (1.2%), human resources management (4.9%), literature (0.7%), marketing (1.0%), psychology (17%), sociology (3.4%) and veterinary science (1.7%).

### Procedures

The application of the inventory’s final version followed the same procedure used in the exploratory study, which means that data collection was also done in approximately ten-minute sessions held in a classroom.

Once again, the participants were informed that they had the option of providing their e-mail addresses to receive a report covering their results, should they wish to do so. After performing all tasks, the collected data were stored in a specific domain created for this purpose and were subsequently analyzed using the statistical software SPSS (version 27), AMOS (version 22) and WINSTEPS (version 5.1.0; [68]).

### Results

#### Construct validity

Firstly, the purpose was to understand the internal structure of the measures and to identify the dimensions and indices associated with it [55]. In order to confirm the results obtained in the exploratory study, a confirmatory factor analysis (CFA) was performed to test whether the measured variables provided an adequate representation of the number of constructs obtained and whether or not the latent factors were responsible for the behavior of the manifest variables [69].

The validation of a model consists of determining its adjustment level towards the available data. In this context, goodness-of-fit measures are particularly important, considering that those indicate the degree to which the correlation matrix—or the variance–covariance matrix—obtained by the model under study reproduces the population matrix. As such, an evaluation regarding the adjustment of the models was carried out using the following measures: chi-square (χ^2^), standardized root mean square residual (SRMR), goodness of fit index (GFI), adjusted goodness-of-fit index (AGFI), incremental fit index (IFI), Tucker-Lewis index (TLI), comparative fit index (CFI), root mean square error of approximation (RMSEA), Akaike information criterion (AIC) and the expected cross-validation index (ECVI).

The structural validity of the skills inventory was tested comparing the two models recommended by the literature review, namely: the iceberg model (two factors; [43] and Great Eight Model (eight factors, [45]).

It can be seen by looking at Table 5 that the model showing a poorer adjustment to the data is the two-factor model, with the covariation of errors suggested by the AMOS modification indices, indicating a high AIC (402.151) and ECVI (2.757) rate. According to Marôco [69], through the calculation of the modification’s indices, it is possible to re-specify the model so that the adjustment improves, as long as the changes made based on these indices are theoretically supported.

**Table 5:** Results of the confirmatory factor analysis for each of the studied models

	*χ*^2^/df	SRMR	GFI	AGFI	IFI	TLI	CFI	RMSEA	LO90	HI90	AIC	ECVI
Two factors model	1.201	0.053	0.893	0.868	0.963	0.958	0.962	0.034	0.011	0.050	411.878	2.813
Eight factors model	1.086	0.051	0.898	0.863	0.983	0.978	0.982	0.022	0.000	0.040	395.200	2.298
*Covariation of errors suggested by the AMOS modification indices*												
Two factors model	1.145	0.052	0.898	0.874	0.983	0.978	0.973	0.029	0.000	0.046	402.151	2.757
Eight factors model	1.072	0.041	0.900	0.864	0.986	0.982	0.985	0.020	0.000	0.039	393.979	2.291

**Fig. 5:**
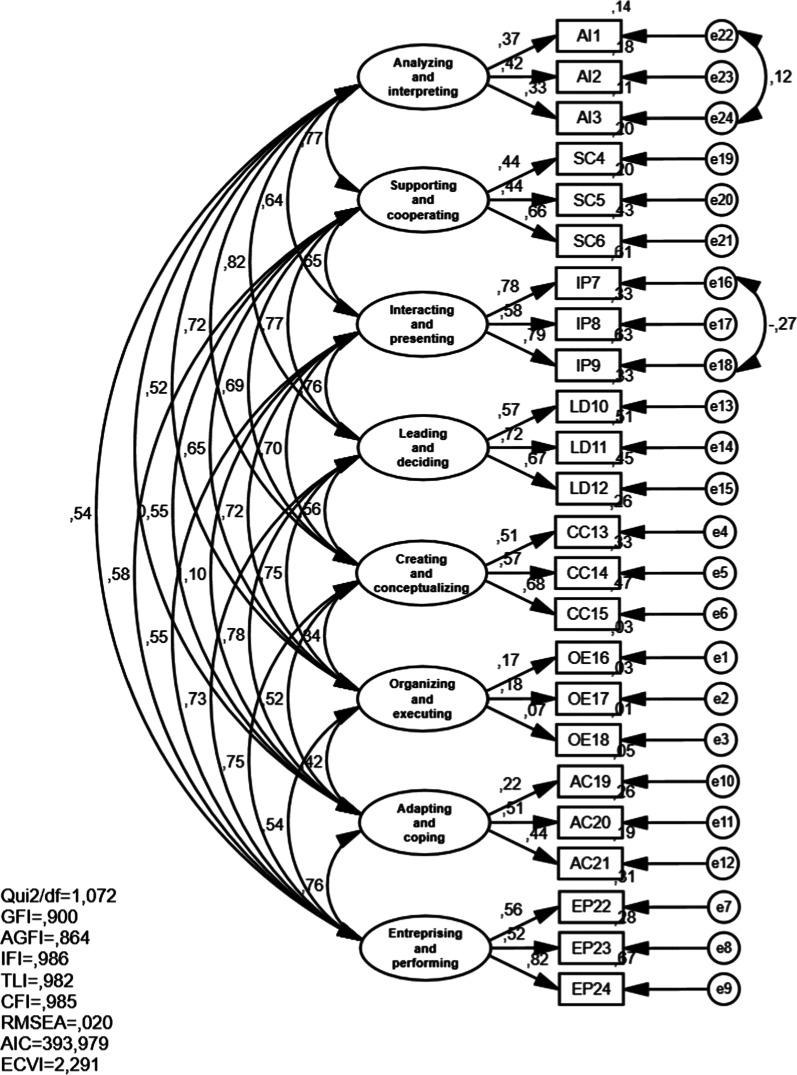
Confirmatory Great Eight model (eight factors)

The results also demonstrate that the eight-factor model, despite having a low AGFI (0.864), is a better fit for the sample data (Fig. 5).

Moreover, it is possible to observe the existence of positive covariations between the eight dimensions, which reveal satisfactory results with respect to the construct’s validity.

#### Reliability

Reliability was analyzed using Cronbach's alpha, following the same steps used in the previous study. Table 6 also reports the measures resulting from the Rasch models, among which we can find the person separation reliability (PSR) coefficient, corresponding to the latent measures for individuals’ variance on the variance of the measures estimated for the same individuals. A satisfactory value of PSR is similar to the value of Cronbach’s alpha co-efficient, ranging from 0.70 to 0.95 [70]. However, a useful reformulation of the PSR as the Person Separation Index (PSI) provides further information about the reliability of a test. It is equal to √r/(1 − r). The higher the value (> 2) of the PSI the more the skills can be differentiated [71, 72]. Observing Table 6, it is possible to see that, with the exception of Enterprising and performing, all skills have values greater than 2, which suggests that all skills are distinguishable from each other. Furthermore, the item separation reliability (ISR), was accounted for, and it corresponds to the percentage of variance of the item not explained by the measurement error [73].

**Table 6:** Reliability indicators for the skills under study

Skills	*Cronbach alpha*	PSR	PSI	ISR
Analyzing and interpreting	0.81	0.81	2.06	0.97
Supporting and cooperating	0.88	0.86	2.70	0.97
Interacting and presenting	0.82	0.80	2.13	0.78
Leading and deciding	0.85	0.84	2.38	0.98
Creating and conceptualizing	0.80	0.80	2.00	0.95
Organizing and executing	0.81	0.81	2.06	0.81
Adapting and coping	0.86	0.85	2.47	0.95
Enterprising and performing	0.73	0.74	1.64	0.99

Despite the PSR values being slightly lower, all of them are above 0.70, which suggests an adequate internal consistency. It was also found that the item separation reliability (ISR) indices are very close to one, which reveals that these measures are reliable [68].

#### Normality

To analyze multivariate normality, the Mardia multivariate kurtosis coefficient was used, as well as its respective critical ratio, and it was found that the data do not follow a normal distribution, since the value of the normalized Mardia coefficient is higher than 10. In Kline's perspective (2005, p. 272), this indicates “a serious violation of the assumption of normality”.

The assumption of normality in the sample data is a required condition for making valid inferences but, according to Klem [74], this is an assumption that rarely occurs in social sciences. It is known that the chi-square fit statistic is often positively biased using the maximum likelihood estimator when the data are nonnormal [75]. Given this, bootstrapping techniques with the Bollen-Stine method [76] appear to be an ideal means to tackle these problems. The covariance structure model on the sample data can study the bootstrapping performance of the fit statistic under the “null hypothesis” that the model fits. The results show when testing the null hypothesis that the model is correct (Bollen-Stine bootstrap: *p* = 0. 637).

#### Dimensionality, Rasch item fit and item difficulty

Through IRT, the authors sought to know the characteristics of the observed variables (items/tasks), and also to estimate the performance of the participants regarding the latter. This approach proposes statistical-mathematical modeling for the latent characteristics of the individual and for the parameters associated with the items [77].

The statistical techniques of IRT have been recognized as being robust strategies to build and validate psychometric instruments, as they allow the verification of the measurement invariance and of the measurement error per item [78]. The Rasch model in particular allows us to calculate the adjustment of the data and to find out whether they deviate from the model, which makes it possible to compare expected and observed results [68].

When considering Rasch models, there are two fundamental indicators in the adjustment statistics of items and subjects to the model: the infit index and the outfit index. The outfit adjustment is more sensitive to outliers and is able to reach high values arising from unexpected responses. The infit adjustment is more robust and, as such, is considered to be the best indicator of the item’s psychometric qualities. For both indicators, the values assume a distribution between 0.00 and infinity, with an expected average value of 1.00 [71].

The data presented in Table 7 demonstrate that the MNSQ and ZSTD values of the items belonging to each dimension are within the reference intervals recommended by Bond and Fox [71] and Tavakol and Dennick [72]. This suggests that the level of difficulty of the items is suitable for the sample under study. It can also be observed that the correlation values are all above 0.40 and below 0.85, which reveals the inexistence of problems, as all the questions were well understood, without any misunderstandings, by the participants [79].

**Table 7:** Item difficulty, standard error, infit and outfit statistics and correlations

			Infit		Outfit		
Skill/Item number	Item difficulty	SE	MNSQ	ZSTD	MNSQ	ZSTD	PTMEA
*Analyzing and interpreting*							
AI1	0.29	0.18	1.05	0.40	1.08	0.60	0.74
AI2	1.09	0.18	0.99	0.01	1.01	0.10	0.73
AI3	− 0.37	0.19	1.07	0.60	1.10	0.70	0.63
*Supporting and cooperating*							
SC4	0.78	0.16	1.21	1.50	1.16	1.20	0.69
SC5	− 0.17	0.17	0.89	− 0.70	1.06	0.40	0.65
SC6	− 0.12	0.17	0.76	− 1.80	0.73	− 2.00	0.75
*Interacting and presenting*							
IP7	0.46	0.14	1.24	1.70	1.18	1.40	0.60
IP8	− 0.32	0.15	1.27	1.80	1.18	1.30	0.60
IP9	− 0.30	0.15	1.01	0.10	0.91	− 0.60	0.74
Leading and deciding							
LD10	− 0.25	0.18	1.26	1.80	1.16	1.20	0.68
LD11	0.81	0.18	0.86	-1.00	0.86	− 1.10	0.76
LD12	− 0.48	0.18	0.79	− 1.70	0.74	− 2.00	0.85
*Creating and conceptualizing*							
CC13	− 0.46	0.19	1.12	1.00	1.10	0.70	0.61
CC14	0.05	0.18	1.05	0.50	1.06	0.50	0.65
CC15	− 2.20	0.23	1.03	0.20	0.84	− 0.50	0.49
*Organizing and executing*							
OE16	2.15	0.15	1.22	1.50	1.20	1.40	0.51
OE17	1.47	0.15	1.15	1.10	1.14	1.10	0.63
OE18	− 2.11	0.21	1.09	0.60	0.86	− 0.40	0.51
*Adapting and coping*							
AC19	− 0.77	0.18	0.92	− 0.60	0.84	− 1.00	0.67
AC20	− 0.46	0.17	0.89	− 0.80	0.89	− 0.70	0.69
AC21	1.39	0.15	0.75	− 1.90	0.79	− 1.60	0.68
*Enterprising and performing*							
EP22	1.94	0.13	1.10	0.80	1.07	0.60	0.66
EP23	0.10	0.14	1.06	0.50	1.04	0.30	0.67
EP24	0.48	0.14	0.86	− 1.10	0.86	− 1.00	0.76

Item difficulty measured in logits (negative values indicate easier questions)

*SE* Standard error, *MNSQ* mean square (values between 0.70 and 1.30 are within acceptable limits for the Rasch model), *ZSTD* value of t-test (values between − 2 and + 2 are within acceptable limits for the Rasch model), *PTMEA* correlations (values between 0.40 and 0.85 are within acceptable limits for the Rasch model)

The analysis of the dimensionality evidences the validity based on the instrument's internal structure, which allows to properly interpret the obtained results [80]. Following this, Alavi and Bordbar [78] state that when infit and outfit values range from 0.70 to 1.30 and do not exceed the value of 2 considered by Linacre [81] as the maximum limit for acceptance of an item, as it interferes with the validity of the test, there is an adequate adjustment of the data for the sample under study.

## Discussion

When considering an ever-changing organizational backdrop, it is increasingly important for organizations to select the best possible talent, as human potential is what allows them to make a difference in an increasingly competitive market [82]. Being employed in today’s labor market is a major challenge. It requires a set of skills that allow for the integration ability and adaptation process to take place amongst constant changes. As such, more than developing technical skills, it is essential to also develop a wide range of soft skills which add value when starting one’s professional career [7]. Particularly, in order for university students to be prepared for the challenges that the labor market offers them, it is essential that they know what is expected of them, namely which skills are employers looking for [37]. With this knowledge these individuals, when looking for their first job, become able to know which roles to adopt, how to manage conflicts, how to coordinate their work and carry out it in a more cooperative and integrated way with peers, superiors and clients [36].

Given this, within this scope, it is essential to choose scientifically developed tools that stand out through their methodological rigor and broad theoretical foundation [83]—otherwise, the entire selection process may be compromised [84].

The development of such an instrument encompasses a continuous technical and conceptual process, which leads to a set of procedures that must ensure the accurate representation of the measurable construct using the items to be included in the inventory [84]. To do so, it was necessary to carry out several complementary analyzes that allow: (1) identification of the test’s structure (factors specified through the EFA and/or CFA; (2) study of the internal consistency (e.g., Cronbach's alpha coefficient, total-item or item-to-item correlations; (3) analysis of the homogeneity of the content of each dimension; and (4) confirmation of the psychometric properties of the instrument in independent samples [85]. Taking this into account, the development of the short skills inventory took place over three studies, whose main conclusions now present.

Study 1 was dedicated to the construction and development of the items, which were subsequently the subject of a spoken reflection. That step revealed a very complete and appropriate inventory to select people in a work context, as it presents a broad theoretical foundation and suitable psychometric indicators. This content analysis and appreciation of the items was extremely enriching, as the suggestions given allowed the items to be readjusted and improved.

The aim of the second study, with its exploratory nature, was to carry out an evaluation of the instrument’s properties using CTT. Data analysis revealed that the instrument ensures the item’s representativeness and adequacy for the measurable construct, with the finding of a factorial structure whose dimensions explain 83.24% of the total variance—a very satisfactory result, considering that values above 50% are deemed as acceptable [86]. These values are much higher than those found by Candel et al. [87] in their study on the influence of the Great Eight Model skills regarding professional performance, which, in turn, obtained an explained variance of 48.08%. The reliability of the inventory was calculated using Cronbach's alpha coefficient, which presented very adequate values [88] for all dimensions, with values ranging from 0.77 to 0.90.

Using the method proposed by Fornell and Larcker [64], it was possible to ascertain that the inventory of short skills of students searching for their first job has an adequate convergent and discriminant validity. Except for the “organize and execute” skill (AVE = 0.48), all AVE were equal to or greater than 0.50 and present values higher than the MSV and the AVS. Through the analysis of the CR it was possible to see that the inventory has a good reliability and convergent validity, with values greater than 0.70 [66].

Furthermore, the existing correlations between the various skills of the Great Eight Model were studied. It was found that there is a significantly positive correlation between all of them, with coefficients ranging from 0.356 to 0.787, with the highest correlation being that between “supporting and cooperation” and “adapting and coping”. According to Sundstrom et al. [67] high levels of these skills are significantly associated with a higher performance in teamwork.

The third study, of a confirmatory nature, was intended to test the studied model comparing it to two theoretical models: The iceberg model [43] and the Great Eight Model [45]. The data analysis revealed that the model under study fits the sample data. Regarding reliability, it was found that all dimensions show very adequate Cronbach's alpha coefficients [89]. It should be noted that, as expected, the PSR indices are slightly lower, but still higher than 0.70, which suggests that the location of items and people on the latent variable scale is replicable [68].

Following the study of the various instruments’ psychometric indicators, a sensitivity analysis was conducted through the use of the Mardia multivariate kurtosis coefficient, which revealed that the data do not follow a normal distribution [90]. Given this, bootstrapping techniques with the Bollen-Stine method were used, and revealed that the model fits.

Regarding dimensionality, it was found that the items have adequate infit and outfit indices [68], given that the percentage of people with results that do not fit the model is low, with values ranging from zero to 13%, which confirms the adequacy of the items to the dimensions being studied [91]. Furthermore, the Point Measure Correlation (PTMEA), also, indicates the respective item can achieve its goals of measuring the construct that needs to be measured [92].

### Theoretical and practical contributions

The construction of this short skills inventory brings several benefits, both at a theoretical and practical level, as it contributes to reducing Portugal’s gap regarding psychological assessment in the selection context [93], considering the lack of validated and verified instruments that fit students looking for their first job [15].

It ensures a greater level of objectivity in the assessment process, thus allowing the inconveniences associated with a poorly managed selection process to be reduced. Inadequate selection involves not only choosing candidates with less potential, but also not hiring really competent individuals, who then become available for competitors [84].

The results found through the analyses carried out over the various studies, show that this instrument has quite satisfactory psychometric properties, and is, as such, a valid and reliable instrument, presenting an alternative to the instruments currently used in the recruitment and selection processes.

Its main advantage regarding other instruments is its size (24 items), as this allows a considerable reduction in application time and also eliminates the undesirable effects of stress and fatigue, which often skew candidates' responses. This is important to mention because the most used skills inventories in the context of selection in Portugal are the CompTEA [94] which includes 172 questions, and the BIP [95] which comprises 220 questions. Furthermore, in a more practical sense, the creation of this inventory allows a faster and more efficient selection process because, as it automatically provides a report with the results, the costs inherent to the administration and quotation of the tests are reduced. Additionally, it also allows comparison of the performance between the various candidates, enabling fairer and more informed decisions. According to Skinner and Pakula [96], this type of instruments represents the time consumed by the process being reduced by around 50.0–75.0%, an extremely important advantage when considering a large-scale people selection operation. Lastly, and in line with Ramadan and Aleksandrovna [77], students looking for their first job tend to be more honest in computerized selection tests and inventories than in traditional pencil-and-paper tests and inventories.

### Limitations and future studies

Regarding the limitations faced during the present study, the fact can be reported that a convenience sample was used, which brings the disadvantage of making it impossible to make inferences at the population level. It is also important to mention the challenges inherent to the availability of rooms with computers and/or internet access, which made it impossible to collect data and obtain a larger sample. Likewise, it is also worth noting the lack of studies that evaluate the predictive validity of the inventory in the work context and that analyze the performance indicators through the key performance indicators methodology, in order to verify the extent to which the results achieved are performance predictors for the selected candidates.

It would also be important to have created an item bank, that allowed the random selection, among a set, of the items that would be used in each situation, thus avoiding the constraints associated with the candidates becoming familiar with such items in future selection processes. On the other hand, it could be interesting to submit the results to several weighting equations, in order to estimate the subject's subsequent performance in various professional areas, and to rank it alongside that of the other candidates [97].

This study only focused on the construct validity, so it would be important to assess aspects pertaining to convergent-discriminant validity [55]. Also, it would be relevant to analyze the Differential Item Functioning (DIF) to see if the answers given to the items are different between different groups. According to Alavi and Bordbar [78] DIF procedures are very useful to determine if individual items in a given skill work in the same way for two or more groups under study (e.g., gender, age, educational qualifications). Taking the gender of the participants as an example, it would be interesting to see if a given item has different levels of difficulty for men and women, that is, if a man and a woman with the same skill level have different probabilities of answering the item correctly [98]. Likewise, studies should not be neglected that correlate the results obtained through the skills inventory during the selection process, with objective measures resulting from the performance evaluation after a year of effective work, in order to assess the predictive validity of the instrument in the workplace.

Considering its characteristics, this instrument may be of considerable use to the scientific research and psychological assessment fields, so it would be interesting to find out more regarding its applicability in a vocational guidance and counseling context among young people who are seeking to start their professional life.

Despite its limitations, this inventory represents a promising instrument regarding people selection, as it brings considerable advantages that come from the gains leveraged from the reduction of selection errors, which represent high costs for organizations—both in the medium and long run.

## Conclusion

The development of an individual from a certain moment in his life is directly related to his entry into the labor market, and from that moment on he will be permanently conditioned by it. Therefore, it is important to know which skills these young individuals need to perceive as essential in order to feel more confident and successful in their workplace.

Furthermore, the systematic increase in the use of computers within the psychological assessment and selection of young and new individuals has been encouraged by technological progress, particularly by: (1) the transformation of paper-and-pencil format instruments into computerized versions; (2) and the evaluation of more complex constructs such as competences [99]. According to Ramadan and Aleksandrovna [77], the increased demand for computerized instruments results from the contributions associated with the conditions of application, the processing of responses and their interpretation. Campion et al. [13] add that some of the main advantages are: reduced application time, lower cost, greater reliability in responses, possibility of feedback with the candidate, creation of a database, standardization, and automatic correction and storage of data.

However, in Portugal, it seems that the number of studies regarding the psychometric characteristics of inventories that allow us to know more about the skills that allow young students looking for their first job is still meager. This research thus aims to help fill in this gap, through the construction and validation of a short skills inventory for this population.

In light of the above, the authors are able to affirm that the short skills inventory, developed within the scope of this investigation, presents reasonably satisfactory psychometric properties [68], thus representing a valid and precise tool that may bring numerous advantages for the recruitment and selection processes for students looking for their first job.

The results found here demonstrate that this inventory is a valuable asset for people selection, and that it may be an alternative regarding the instruments currently in use. Its innovative character, in addition to the increased satisfaction and motivation of the candidates during the tests, allows for the reduction of errors inherent to the administration and quotation of the tests [100].

The selection process is, as such, no longer seen as a cost factor and is now considered a decisive investment for an organization to achieve its objectives and fulfill its needs, both in the short and in the medium-long term [84].

## Data Availability

The datasets generated during and analysed during the current study are available from the corresponding author on reasonable request.

## Abbreviations

AGFI: Adjusted Goodness-of-Fit Index
AIC: Akaike Information Criterion
ASV: Average Shared Square Variance
AVE: Average Variance Extracted
CFA: Confirmatory Factor Analysis
CFI: Comparative Fit Index
CR: Composite Reliability
CTT: Classic Theory of Tests
DIF: Differential Item Functioning
ECVI: Expected Cross-Validation Index
EFA: Exploratory Factor Analysis
GFI: Goodness of Fit Index
IFI: Incremental Fit Index
IRT: Item Response Theory
ISR: Item Separation Reliability
KMO: Kaiser–Meyer–Olkin
M: Mean
MSV: Maximum Shared Square Variance
PCA: Principal Components Analysis
PSI: Person Separation Index
PSR: Person Separation Reliability
PTMEA: Point Measure Correlation
RMSEA: Root Mean Square Error of Approximation
SD: Standard Deviation
SRMR: Standardized Root Mean Square Residual
TLI: Tucker-Lewis Index
χ^2^: Chi-Square

## Footnotes

1 Hereinafter, also referred to as “skill”.

2 Partner A.

3 Partner B.
